# RNA sequence analysis of dermal papilla cells’ regeneration in 3D culture

**DOI:** 10.1111/jcmm.15965

**Published:** 2020-10-10

**Authors:** Guanyu Lin, Guoqian Yin, Jun Ye, Xinyuan Pan, Jiangying Zhu, Bojie Lin

**Affiliations:** ^1^ Department of Plastic and Aesthetic Surgery The First Affiliated Hospital of Guangxi Medical University Nanning China; ^2^ Department of Emergency Surgery The Affiliated Zhuzhou Hospital Xiangya Medical College CSU Zhuzhou China; ^3^ Department of Plastic and Aesthetic Surgery The People's Hospital of Guangxi Zhuang Autonomous Region Nanning China

**Keywords:** 3D culture, dermal papilla cells, hair follicle, regeneration, RNA‐seq

## Abstract

It is well known that dermal papilla cells (DPCs) are crucial for hair follicle growth and regeneration. However, dermal papilla cells in 2D culture could lose their ability of regeneration after several passage intervals. As opposed to DPCs in 2D culture, the DPCs in 3D culture could passage extensively. However, the molecular mechanisms of DPCs’ regeneration in 3D culture remain unclear. Accordingly, gene sequencing is recommended for the investigation of hair regeneration between 2D and 3D culture, the three groups were established including DPCs in passage 2 in 2D culture, DPCs in passage 8 in 2D culture and DPCs in passage 8 in 3D culture. The differentially expressed genes (DEGs) were identified using the Venn diagram of these three groups, which included 1642 known and 359 novel genes, respectively. A total of 1642 known genes were used for Gene Ontology (GO), Kyoto Gene, Genomic Encyclopedia (KEGG) pathway enrichment and protein‐protein interaction (PPI) analyses, respectively. The functions and pathways of DEGs were enriched in biological regulation, signal transduction and immune system, etc. The key module and the top 10 hub genes (*IL1B, CXCL12, HGF, EGFR, APP, CCL2, PTGS2, MMP9, NGF* and *SPP1*) were also identified using the Cytoscape application. Furthermore, the qRT‐PCR results of the three groups validated that the hub genes were crucial for hair growth. In conclusion, the ten identified hub genes and related pathways in the current study can be used to understand the molecular mechanism of hair growth, and those provided a possibility for hair regeneration.

## 1 INTRODUCTION

Hair follicles are actively involved with thermoregulation, protection and adaptive coloration of human species; hence, the rapid loss of hair has received enormous attention from researchers worldwide.^1^ Typically, the hair follicle includes the cuticle and epithelial cells, which constitute the hair shaft, inner and outer root sheaths, dermal papilla and matrix progenitor, respectively. The dermal papilla (DP) is a unique population of the mesenchymal cells which instruct the keratinocytes in the follicle to generate hair shafts.^2^ Although most studies reported that dermal papilla is responsible for hair generation, its exact mechanism is not clear.^3^, ^4^

Cell culture technology is vital for the study of diseases and cancer. Currently, the three‐dimensional (3D) culture technology is rapidly growing in the life science sector. As compared to 2D culture, cells in 3D culture technology are closer to the complex in vivo conditions.^5^ A significant advantage of 3D culture over 2D culture conditions is the decrease in the gap between cell models in vitro and in vivo. Besides, the features of 3D culture cells are closer to the complex in vivo condition.^6^ Some researchers found that 3D cell cultures could induce pluripotent stem cells (iPSC) to generate personalized tissue models.^7^ As the dermal papilla cells (DPCs) are unique mesenchymal cells, we previously found out that DPCs could also induce human hair growth in mice by transplanting intact DPCs and epithelial cells.^4^, ^8^ However, such a process requires large numbers of human DPCs as they lose the ability of hair inductivity in 2D in vitro culture. Therefore, most researchers reported that the 3D‐spheroid culture of DPCs could restore DPCs' inductive characteristics; however, the science behind such observations is unknown.^9^ RNA‐Seq is an excellent approach to study transcriptome profiling using deep‐sequencing technologies.^10^ The transcriptome includes all sets of transcripts in a cell, which is crucial for further analysis of its molecular constituents and gene functionality. The objective of transcriptomics is to categorize all species of the transcript, which includes mRNAs, LncRNAs, CircRNAs and MicroRNAs.^11^ RNA‐Seq provides a new approach to analyse eukaryotic transcriptomes using a novel, high‐throughput and cheaper DNA sequencing methods than traditional methods such as microarray technology^12^ and Sanger sequencing technology.^13^ Therefore, in this study, we applied the novel RNA‐Seq technology to investigate the effect of 3D culture on reversing the ageing process of DPCs. Gene Ontology (GO), Kyoto Gene and Genomic Encyclopedia (KEGG) pathway enrichment analyses, as well as protein‐protein interaction (PPI) network analysis, were conducted on genes to identify the molecular processes of DPCs’ development and progression in 3D culture as compared to 2D culture.

## 2 METHODOLOGY

### 2.1 Human dermal papilla cells in 2D culture

Intact human dermal papillae were isolated from hair follicles in occipital scalp during hair transplantation surgery of patients. We removed the matrix and exposed the dermal papilla from the follicles by fine needles. Papillae were isolated by cutting through their stalk. Next, 6‐8 papillae were cultured in 35‐mm dishes containing 20% foetal calf serum (FBS) in Dulbecco's modified eagle medium (DMEM) with 1% three antibiotics medium (penicillin, streptomycin and fungizone). Papillae were adhered to the bottom of the culture dish by fine needles. We observed that cells could migrate from the papillae, and the intact papillae eventually collapsed after 7 days. When dermal papilla cell density approached 80% confluence, the cells were passaged at 1:2 using trypsin containing 0.5% ethylene diamine tetraacetic acid (EDTA). These cells were passaged twice and tagged as Passage 2 in 2D culture (P2‐2D group). The dermal papilla cells were passaged 8 times, and therefore, the cells were named Passage 8 in 2D culture (P8‐2D group). The dermal papillae cells were from four donors, so each group contained four repeated samples.

### 2.2 Human dermal papilla cells in 3D culture

GravtityPlus hanging‐drop plates were used for 3D culture. The plates consisted of the following components: (a) The bottom plate with a reservoir. (b) GravityPLUS Plate (raster plate) with 12x8‐well strips. (c) Lid. (d) Channel—for evaporation‐control liquid. (e) SureDrop hanging‐drop 8‐well strips. (f) Humidifier pads (delivered in bags with 5 pcs with tweezers). According to the manufactures' protocol, the preparation steps were as follows: (a) GravityPLUS bag was wiped with 70% EtOH before opening. (b) The bag was opened under sterile working conditions, and GravityPLUS Plates assembly was taken out. (c) The humidifer pads were removed from the bag using tweezers and placed inside the reservoir. The pads were soaked in 15 mL of 0.5 × PBS. GravityPLUS plates were placed at the bottom plates. (d) Trypsinize cells were observed to expand in cell culture flasks according to the standard protocol. (e) The cells were counted according to previous studies (2500 cells/40 μL).^4^ Next, the Hanging‐drop formation was as follows: (f) 40 µl of cell suspension were delivered to each well on the GravityPLUS plate. A tight contact between the pipette tip and the wells' inlet was established by applying a slight pressure that sealed the environment. (g) Sterile water was added to the outer channel of the GravityPLUS plates for additional evaporation control. (h) The lids were placed on GravityPLUS Plates. (i) GravityPLUS Plate assembly was kept in a humidified CO_2_ incubator at 37°C. (j) The microtissue formation was assessed routinely, and the formation of 3D spheroid was observed after 7 days in the culture. The cells that were passaged 8 times in 3D culture (P8‐3D group) including four repeated samples were used in experiments.

### 2.3 Total RNA extraction and mRNA library construction

P2‐2D group, P8‐2D group and P8‐3D group' RNA were extracted with TRIzol.^14^ Agilent 2100 Bioanalyzer was used to detect the quality of RNA. The cDNA libraries were synthesized and amplified with the supplemented oligo primers. Next, they were loaded onto the flow cell channels of a BGISEQ‐500 platform for sequencing at the Beijing Genomics Institute (BGI), Shenzhen, China.

### 2.4 Identification of differentially expressed genes (DEGs)

The most common limitation of sequence data is the presence of false positives. The presence of false positives can be accounted for by adjusting the *P*‐value (Q‐value) as well as calculating the Benjamini and Hochberg false discovery rate, which also simplifies the identification of statistically significant genes. A logarithmic fold change value (logFC)> 1 and an adjusted *P*‐value <.05 were used to choose the differentially expressed genes (DEGs) among three groups (P2‐2D group, P8‐2D group and P8‐3D group). The overlap of two datasets (between P8‐2D group and P2‐2D group, between P8‐2D group and P8‐3D group) was identified by drawing Venn diagrams.

### 2.5 GO and KEGG enrichment analysis of the DEGs

The Database for Annotation, Visualization and Integrated Discovery (DAVID; version 6.8; http://david.ncifcrf.gov)^15^ is an online database that analyses bioinformatic data with online tools and offers an exhaustive result of the functions of genes or proteins. Kyoto Encyclopedia of Genes and Genomes (KEGG) is used to comprehend the advanced features of a biological system using molecular datasets at the scale at which it is generated by high‐throughput experimental techniques. KEGG (KEGG, https://www.kegg.jp/) is a database that helps users assign related molecular process, diseases and pathways of genes by high‐throughput technology.^16^ Gene Ontology (GO) is an essential web‐based tool for annotating genes and unifying biological processes, cellular components and molecular function of target genes.^17^ The biological function of the DEGs was analysed using the online database, DAVID. A Q‐value (Adjusted *P*‐value) of <.05 was considered to indicate statistical significance.

### 2.6 Construction of a PPI network and analysis of module

Search Tool for the Retrieval of Interacting Genes (STRING; http://string‐db.org) (version 10.5) is an online database to analyse the protein‐protein interaction (PPI) network of genes.^18^ STRING database provides users a valuable spot about molecular mechanisms of genes or the occurrence of diseases via analysis of all functional interactions between the expressed proteins of target genes. In our study, the STRING database was used to construct the PPI network of differentially expressed genes. The data with a combined score of >0.4 was defined as a statistically relevant interaction. Cytoscape (version 3.7.0) is an open‐access bioinformatics software that is used to draw and analyse functional interactions of target genes downloaded from the STRING database.^19^ Furthermore, Molecular Complex Detection (MCODE) (version 1.5.1) is an application plugin in Cytoscape to analyse the PPI network, which can identify densely connected and bipartite network modules of target genes. In this study, the Cytoscape application was used to analyse the PPI network of DEGs; we also applied the MCODE plugin to identify the most crucial modules. The standards that were applied were as follows: degree cut‐off = 2, node k‐score = 2, score cut‐off = 0.2, Max depth = 100 and MCODE scores >5.

### 2.7 Hub genes selection and analysis

CytoHubba is an app plugin in Cytoscape that can obtain hub genes. After the construction of the gene network, the top 10 with degree ≥10 were identified as hub genes.^20^, ^21^, ^22^ The term ‘degree’ is defined as a number in the Cytoscape setting, which represents the relevance of one gene with other genes.

### 2.8 Expression calculation of Hub genes

RSEM application ([17.18]v1.2.8) ^23^, ^24^ was used to calculate the expression of hub genes (3 groups of 4 samples).

### 2.9 Quantitative RT‐PCR

The total RNAs were extracted using the RNAeasy kit (Takara Biotechnology) to validate the top 10 genes' expression levels. The first‐strand cDNA was synthesized with the cDNA Synthesis kit (ThermoFisher Scientific) according to the manufacturer's instructions. Quantitative real‐time PCR (qRT‐PCR) was performed on the CFX96 Detection System (Bio‐Rad Laboratories) according to the manufacturer's protocol. The relative gene expression level was calculated by the ΔΔCt method in three groups.

## 3 RESULTS

### 3.1 Cellular morphology of three groups

**Figure 1:**
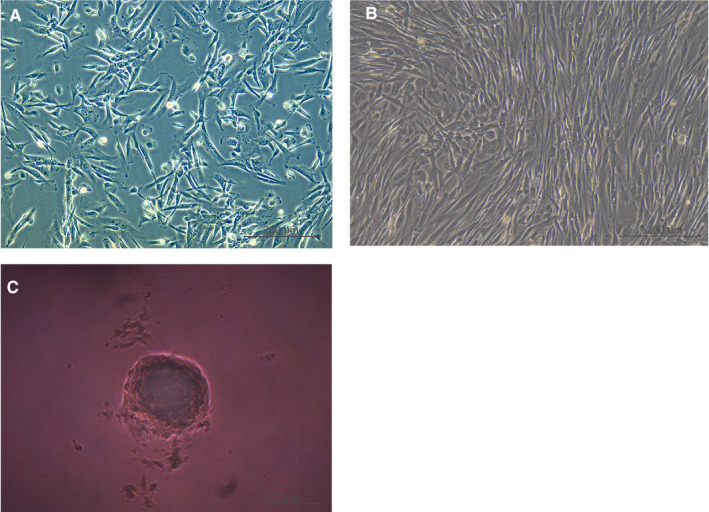
Cellular morphology of dermal papilla cells in three groups (inverted phase‐contrast microscope 10 x 10): (A) P2‐2D; (B) P8‐2D; (C) P8‐3D

**Figure 2:**
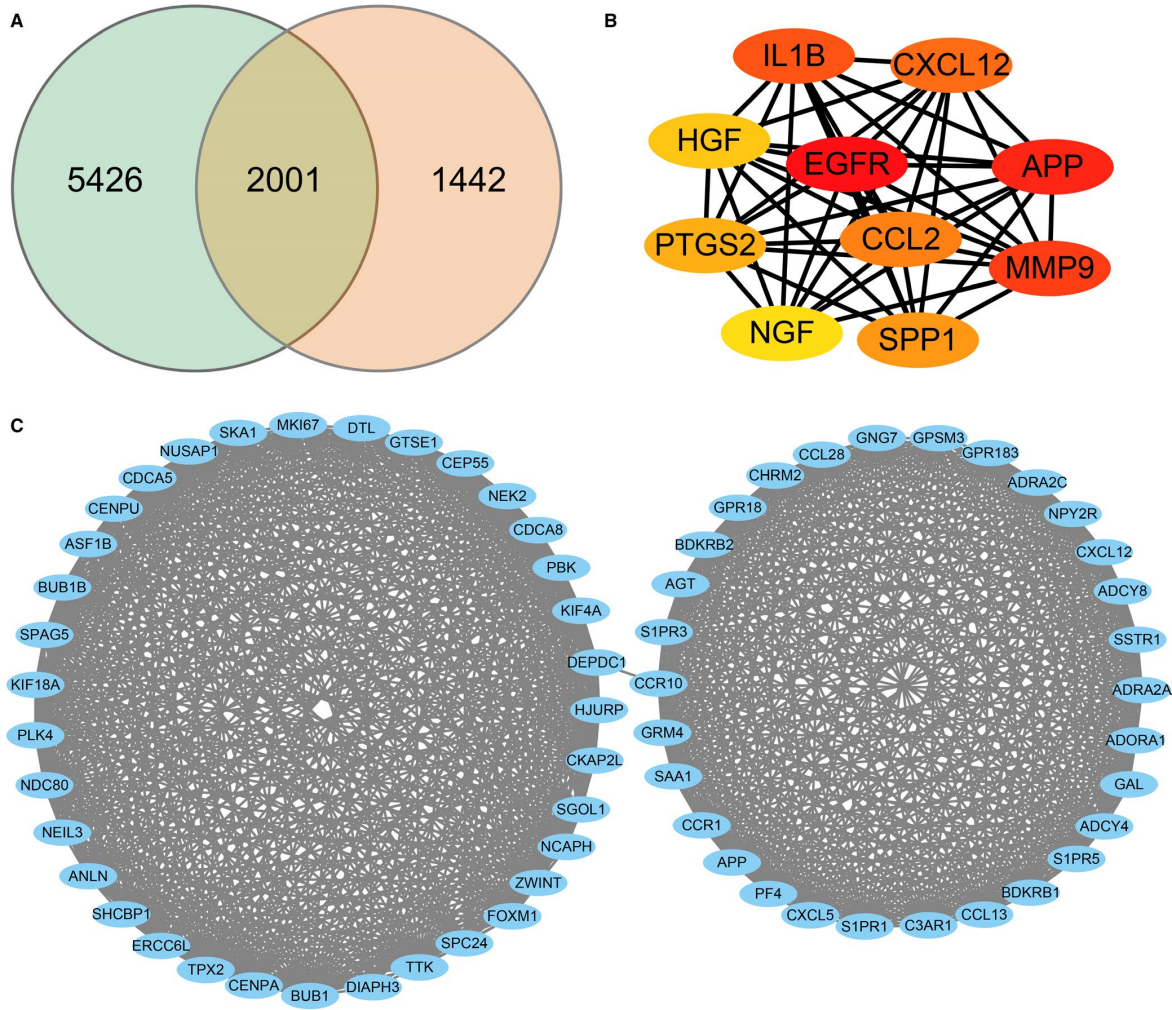
(A) Venn diagram of the two RNA‐seq datasets (7427 of p8‐2D vs p2‐2D and 3443 of p8‐2D vs p8‐3D). The blue circle indicates datasets of p8‐2D vs p2‐2D, and orange circle indicates datasets of p8‐2D vs p8‐3D. DEGs were selected with fold change >1 and Adjusted *P*‐value <.05. (B) PPI network of hub genes with 10 nodes and 44 edges. The colour depth of the nodes represents the degree level. The deeper the colour, the higher is the degree. (C) The module with the maximum relevance was obtained from the PPI network with 65 nodes and 991 edges

When DPCs were passaged twice in the 2D culture, the cells formed a star‐shaped morphology that tends to stick together (Figure 1A). In contrast, the shape of each cell became longer and less concentrated after the DPCs were passaged eight times than those DPCs that were passaged twice in 2D the culture, respectively (Figure 1B). Furthermore, the DPCs aggregated to a multicellular spheroid (diameter: 100‐200 μm) after the DPCs were passaged eight times in 3D culture (Figure 1C). DEGs (7427 in p8‐2D vs p2‐2D and 3443 in p8‐2D vs p8‐3D) were identified following the RNA sequence standardized results (*P* < .05, log|FC|>1). The intersection of the two datasets consisted of 2001 genes including 1642 known genes and 359 novel genes, as shown in the Venn diagram (Figure 2A).

### 3.2 Classification and enrichment analysis of DEGs

**Figure 3:**
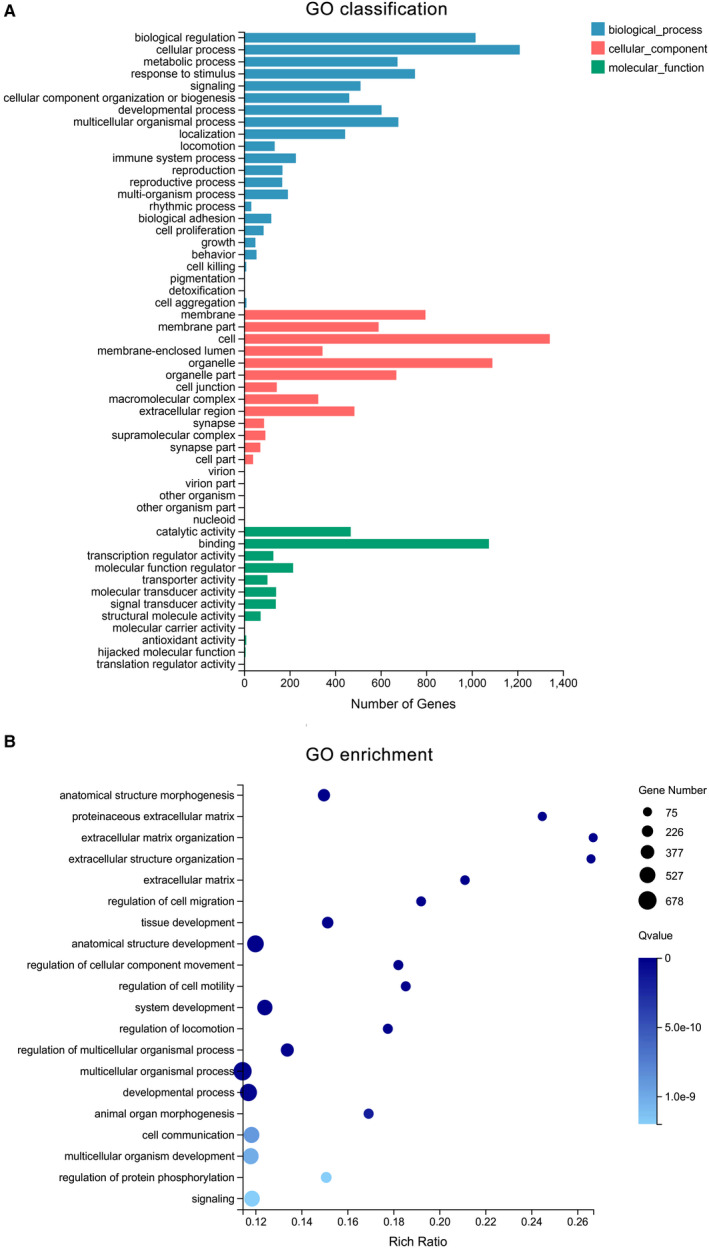
Gene ontology (GO) analysis of DEGs. (A) Blue column means the biological process, whereas the red column means a cellular component, and the green column indicates molecular function. (B) the size of the bubble signifies the gene number; the colour depth represents the Q‐value, whereas the rich ratio indicates the gene number/the total gene number in the y‐axis item

**Figure 4:**
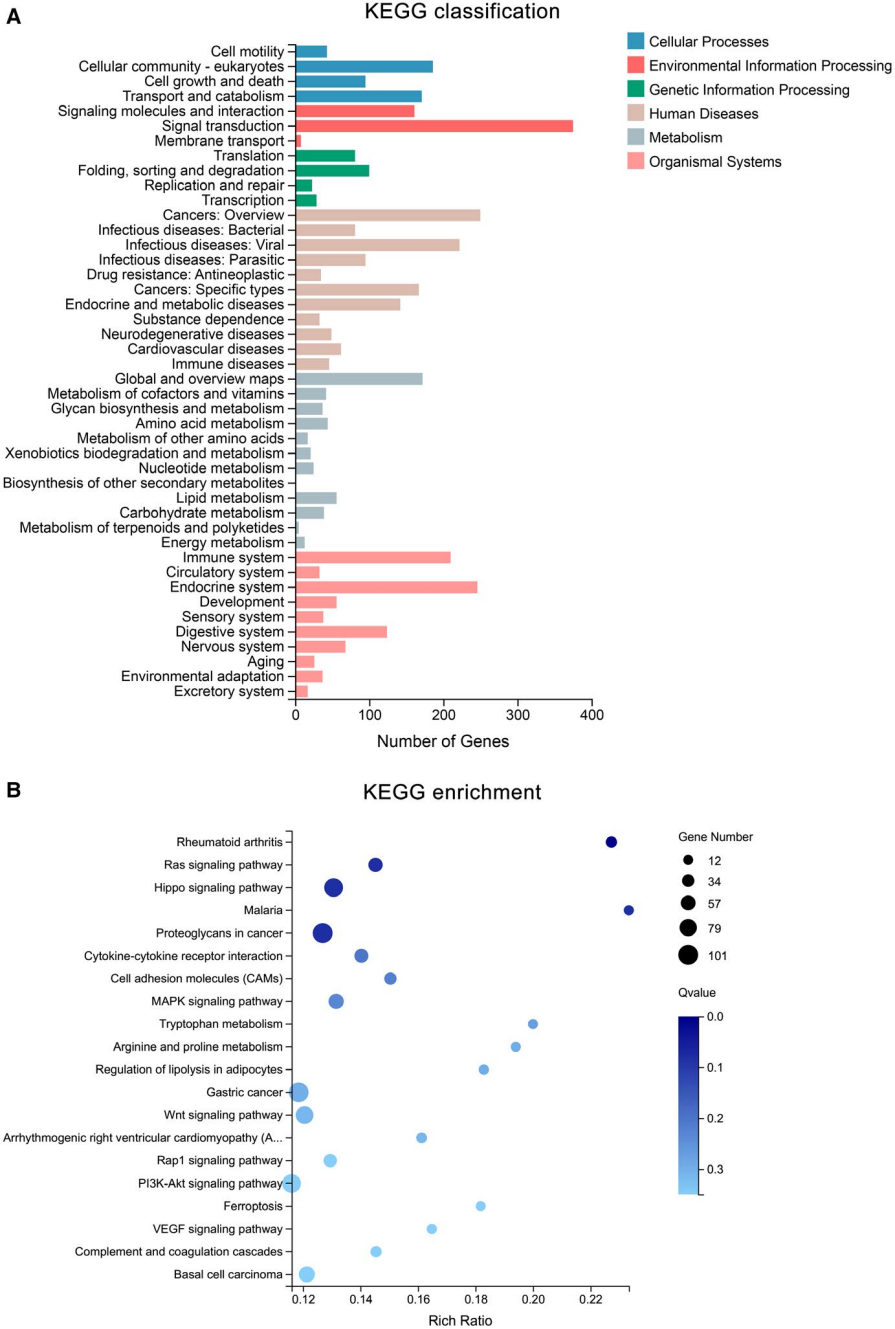
Kyoto encyclopedia of genes and genomes (KEGG) analysis of DEGs. (A) Blue and red columns mean the cellular process and environmental information processing, respectively. The green column indicates the processing of genetic information processing, whereas the brown column represents human diseases. The grey and pink column indicates the metabolism and organismal system, respectively. (B) The size of the bubble means gene number; the colour depth means Q‐value, and the rich ratio means the gene number/the total gene number in the y‐axis item

Gene Ontology and KEGG analysis of the DEGs (1642 known genes), which included the functional classification and enrichment analyses, were performed using the DAVID online tool. The results of the GO analysis showed that alterations of biological processes (BP) in the DEGs were markedly changed during the cellular processes, the biological regulation, and in response to stimuli (Figure 3A). DEGs' alterations in the cell component (CC) were mainly located in cell, membrane and organelle (Figure 3A). The changes in gene functionality at the molecular level (MF) were primarily related to binding, catalytic activity and regulation of molecular activities (Figure 3A). GO enrichment analysis showed that alterations were related with multicellular organismal process, anatomical structure development, developmental process and so on (Figure 3B). Furthermore, DEGs in the KEGG classification were associated with cellular community, signal transduction, immune system, etc (Figure 4A). The KEGG pathway analysis indicated that DEGs were enriched with the Ras signalling, Hippo signalling, MAPK signalling, Wnt signalling, Rap1 signalling, PI3K‐Akt signalling and VEGF signalling pathway, respectively (Figure 4B).

### 3.3 Construction of the PPI network and module analysis

The most significant module with 65 nodes and 991 edges was constructed in Figure 2C by using the Cytoscape MCODE application.

### 3.4 Selection and analysis of Hub genes

Top ten genes with degrees ≥10 were recognized as hub genes. The PPI network of ten hub genes (*IL1B*, *CXCL12*, *HGF*, *EGFR*, *APP*, *PTGS2*, *CCL2*, *NGF*, *SPP1*, *MMP9*) along with 10 nodes and 44 edges were identified in Figure 2B by using the Cytoscape CytoHubba application.

### 3.5 Expression of hub genes

**Figure 5:**
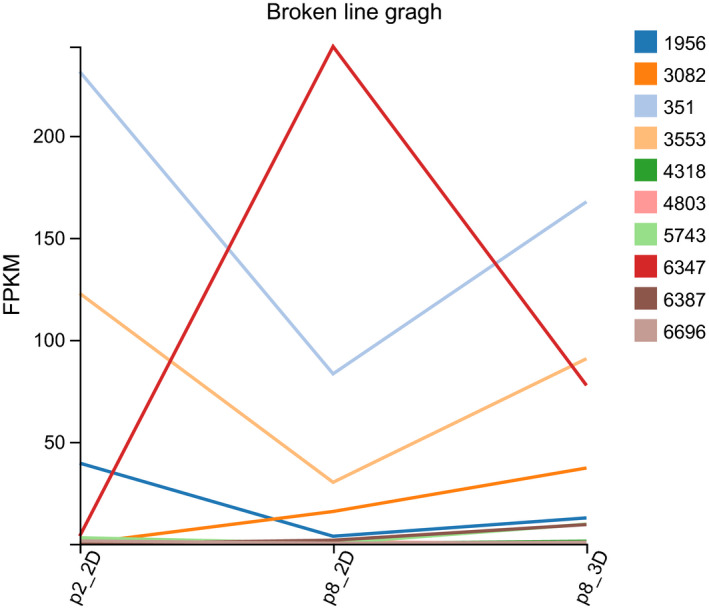
The expression of hub genes. The colour icons mean gene ID (https://www.ncbi.nl m.nih.gov/gene). (1956: *EGFR*, 3082: *HGF*, 351: *APP*, 3553: *IL1B*, 4318: *MMP9*, 4803: *NGF*, 5743: *PTGS2*, 6347: *CCL2*, 6387: *CXCL12*, 6696: *SPP1*) and FPKM (Fragments per Kilobase Million) is an expression of the genes

P2‐2D, P8‐2D and P8‐3D' FPKM (Fragments per Kilobase Million) value of the hub genes are shown in Table 1. The changing trend of hub genes among the three groups is shown in Figure 5. We found out that the hub genes' functionality partially returned to P2‐2D groups' expression level (except for *CXCL12* and *HGF*) for DPCs that were cultured in 3D conditions.

**Table 1:** The FPKM value of hub genes in three groups

Genes	P2‐2D' FPKM	P8‐2D' FPKM	P8‐3D' FPKM
*EGRF*	39.56 ± 1.14	3.73 ± 1.48	12.72 ± 11.25
*HGF*	0.06 ± 0.04	15.86 ± 12.84	37.17 ± 32.87
*APP*	231.50 ± 3.00	83.32 ± 40.12	167.60 ± 84.29
*IL1B*	122.8 ± 0.82	30.17 ± 59.59	90.73 ± 175.70
*MMP9*	1.49 ± 0.11	0.08 ± 0.12	1.32 ± 1.54
*NGF*	0.04 ± 0.02	1.02 ± 0.77	0.11 ± 0.08
*PTGS2*:	2.98 ± 0.12	0.25 ± 0.15	9.66 ± 5.80
*CCL2*	3.80 ± 0.50	243.80 ± 189.00	77.69 ± 7.16
*CXCL12*	0.10 ± 0.04	1.78 ± 2.49	9.46 ± 9.90
*SPP1*	1.42 ± 0.24	0.33 ± 0.46	0.81 ± 0.77

### 3.6 The expressions of qRT‐PCR

**Figure 6:**
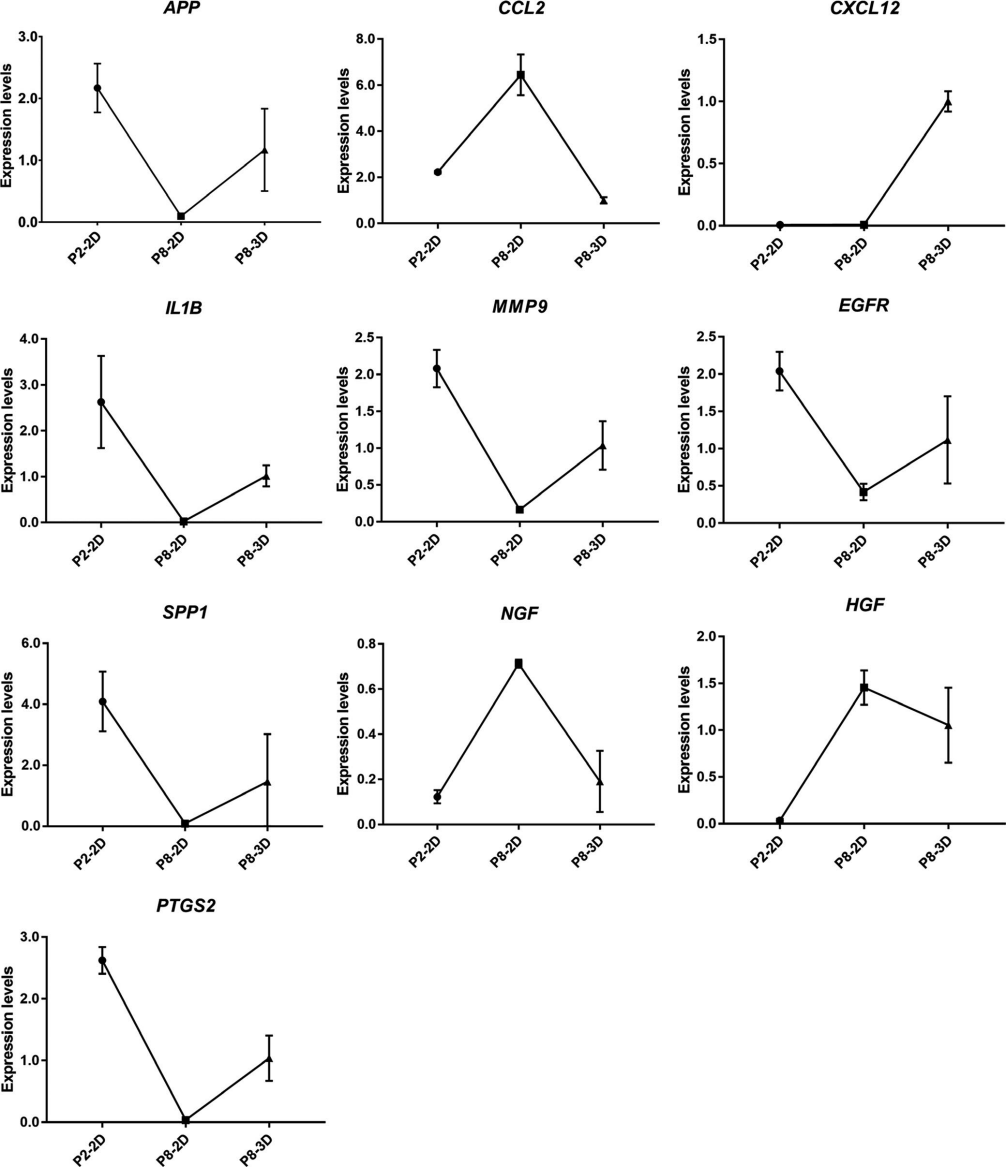
The mRNA expression levels of hub genes. The 2^−⊿⊿Ct^ method was used as calculating mRNA expression levels

Figure 6 showed that the mRNA expression levels of hub genes (top 10) were similar to the RNA‐seq results, that is, the expression levels of hub genes tends to return to the level as P2‐2D groups after DPCs were passaged 8 times in 3D culture.

## 4 DISCUSSION

Recently, the rates of hair loss among adults are on the rise, and limited treatment options are available for such patients.^25^, ^26^ Most studies confirmed that the dermal papilla cells (DPCs) cause ageing of hair follicles.^27^, ^28^ With age, the hair follicle becomes old, and the mitotic capacity of cells worsen. DPCs could facilitate the growth of hair follicle, that is, promote anagen stage and delay the catagen stage of hair follicle *via* releasing exosomes which regulate the stem cells of hair follicles.^29^, ^30^ Researchers also found out that the cycling and morphogenesis of hair follicles could be controlled by DPCs, which require multiple stimulatory and inhibitory signals, such as the Wnt signalling pathway.^31^ DPCs are reported to lose the ability of differentiation after being passaged high times.^4^ However, most studies demonstrated that 3D culture, especially Hangdrop technology, could enhance DPCs' regeneration in hair follicles after multiple passage times by the classical Wnt signalling pathway.^3^, ^32^ Such an observation could be attributed to the extracellular matrix (ECM), one of the critical components of cell growth that has a tremendous impact on the development of hair dermal papilla cells.^33^, ^34^ Nevertheless, the contributions from other pathways are uncertain. With the continuing development of bioinformatics, RNA‐sequencing(RNA‐seq) has become a standard tool to study the molecular mechanism of diseases and species.^35^, ^36^ To the best of our knowledge, we are the first to explore the mRNA expression profiles of DPCs between 2D and 3D culture using high‐throughput sequencing. We identified the top ten hub genes (*IL1B*, *CXCL12*, *HGF*, *EGFR*, *APP*, *PTGS2*, *CCL2*, *NGF*, *SPP1*, *MMP9*) that are typically associated with the 3D culture of DPCs. Consequently, we demonstrated the role of such genes in regulating hair follicle ageing and regeneration.

According to our studies, when DPCs were passaged eight times, the cell morphology of DPCs could transform from star shape to spindle shape; and likewise, the ability of cells to form clusters or colonies decrease. Following the passage of DPCs (8 times) in 3D culture, the cells came together to form a multicellular spheroids, which are similar to the structure of hair follicles' dermal papilla in vivo. Hang drop 3D culture technology induces DPCs to form a spheroid using gravity, which could simulate in vivo conditions. Therefore, we could use hang drop 3D culture technology to restore the cellular function of DPCs, partially. However, the related molecular mechanism is still controversial. Therefore, we divided DPCs into three groups: P2‐2D group, P8‐2D group and P8‐3D group. Each group included four repeated samples to avoid the influence between different individuals. DPCs were offered by four donors, and patients with systemic disease were excluded from this study. The experimental results were verified in different individuals to increase credibility of founding. We acquired the genes (DEGs between P8‐2D group and P2‐2D group) that had changed after DPCs were passaged multiple times. Moreover, we also obtained the DEGs between P8‐2D group and P8‐3D group consisting of genes that had changed when DPCs were cultured in 3D conditions. Next, we made the Venn diagram of two datasets (P8‐2D *vs*. P2‐2D, P8‐2D vs. P8‐3D) to identify 2001 genes including 1642 known genes and 359 novel genes that regulate hair growth. GO and KEGG analysis of 1642 known genes showed that the hair regeneration could be associated with biological regulation, cellular process, metabolic process, response to a stimulus, signalling, immune system process, reproduction, reproductive process, biological adhesion, cell proliferation, membrane, organelle, cell junction, catalytic activity, binding, Ras signalling pathway, Hippo signalling pathway, MAPK signalling pathway, Wnt signalling pathway, Rap1 signalling pathway, PI3K‐Akt signalling pathway and VEGF signalling pathway, respectively. More specially, we found out that a large number of signalling pathway were related to hair growth. Previously researchers tend to choose one signalling pathway, so the results of such studies were usually incomplete. Sirtuin‐1 gene is reported to protect the stem cells of hair follicles *via* the activation of MAPK‐ERK‐Mfn2 pathway.^37^ Hawkshaw et al found out that the Wnt pathway inhibitor could treat human hair loss.^38^ Kim et al indicated that HFSC proliferation could activate the PI3K/Akt pathway.^39^ Li et al^40^ found out that the *VEGF* gene could induce the proliferation of dermal papilla cells through *ERK*. Doma et al^41^ found out that the EGFR‐Ras‐Raf signal pathway played crucial roles in hair follicle development and regeneration. Our results indicated that hair regeneration was connected to the Ras signalling pathway, Hippo signalling pathway, MAPK signalling pathway, Wnt signalling pathway, Rap1 signalling pathway, PI3K‐Akt signalling pathway and VEGF signalling pathway, respectively. The novel point was that Hippo signalling and Rap1 signalling pathways are barely reported to have contributed to hair growth. Therefore, we applied the STRING online tool to analyse the PPI network of 1642 DEGs and used the Cytoscape MCODE application to find the maximum relevance of gene modules with 65 nodes and 991 edges. Finally, our current study found out the top ten hub genes (*IL1B*, *CXCL12*, *HGF*, *EGFR*, *APP*, *PTGS2*, *CCL2*, *NGF*, *SPP1*, *MMP9*) using Cytoscape CytoHubba application. For FPKM value, we found out that the hub genes’ expression levels were different from primary DPCs (P2‐2D groups)when dermal papilla cells were passaged for multiple times (P8‐2D groups). The expression of genes related with cell growth, proliferation, differentiation, transcriptional activation, reproduction and tissue remodelling (*EGRF*, *APP*, *IL1B*, *MMP9*, *PTGS2* and *SPP1*) was decreased. And the expression of genes associated with abnormal differentiation and inflammation (*HGF*, *NGF*, *CCL2* and *CXCL12*) was increased. However, the hub genes returned partially to the level of primary dermal papilla cells (P2‐2D groups) except for *CXCL12* and *HGF* after the DPCs were cultured in 3D conditions (P8‐3D groups). Furthermore, the expression of hub genes in qT‐PCR validated results of FPKM from RNA‐seq.

Therefore, we concluded that 3D culture technology could renew DPCs' ability to induce hair regeneration to a limited extent. According to the hub genes’ function, it could be speculated that the genes of *EGRF*, *APP*, *IL1B*, *MMP9*, *PTGS2*, *SPP1* promoted the hair growth by regulating DPCs' function of cell growth, proliferation, differentiation, transcriptional activation, reproduction and tissue remodelling, and the genes of *HGF*, *NGF*, *CCL2*, *CXCL12* inhibited hair growth by regulating DPCs' function in relation to abnormal differentiation and inflammation. Moreover, direct intervention of hub genes by overexpressing the genes of *EGRF*, *APP*, *IL1B*, *MMP9*, *PTGS2*, *SPP1* and inhibiting the expression of *HGF*, *NGF*, *CCL2*, *CXCL12* might cause permanent regeneration of hair follicles. Novelly, there was barely any report that *SSP1*, *CCL2* and *APP* hub genes could regulate hair growth. The hub genes could be potential biomarkers for the diagnosis and treatment of hair loss. In the future, we attempt to control the expression of hub genes in vitro and in vivo to observe the changes in the morphology of DPCs. Therefore, further research is necessary to verify our findings, which are a limitation to our current study.

In conclusion, the present study identified 2001 DEGs, 10 hub genes and related signalling pathways that were associated with functional recovery of dermal papilla cells in 3D culture. The genes were identified as mechanism of DPCs' regeneration in 3D condition and potential biomarkers to control hair growth.

## CONFLICT OF INTERESTS

There are no conflicts to declare.

## AUTHOR CONTRIBUTION

**Guanyu Lin:** Conceptualization (equal); Data curation (equal); Formal analysis (equal); Investigation (equal); Methodology (equal); Software (equal); Writing‐original draft (equal); Writing‐review & editing (equal). **Guoqian Yin:** Data curation (equal); Formal analysis (equal); Funding acquisition (equal). **Jun Ye:** Investigation (equal); Methodology (equal). **Xinyuan pan:** Writing‐review & editing (equal). **Jiangying Zhu:** Writing‐review & editing (equal). **Bojie Lin:** Conceptualization (equal); Funding acquisition (equal); Project administration (equal); Resources (equal); Supervision (equal); Validation (equal); Visualization (equal).

## Data Availability

All sequence data in this study are available in https://dataview.ncbi.nlm.nih.gov/object/PRJNA616431?reviewer=abkph6a52e4eui7516ltveucv7.
